# Construction and evaluation of machine learning models for postoperative prognosis prediction of biliary tract malignancies

**DOI:** 10.3389/fonc.2026.1918078

**Published:** 2026-09-15

**Authors:** Ji Li, Wei Zhao, Qi He, Jiangyan Che, Chengyou Du

**Affiliations:** 1 Chongqing Medical University, Chongqing, China; 2 Department of Hepatobiliary Surgery, The First Affiliated Hospital of Chongqing Medical University, Chongqing, China; 3 the People’s Hospital of HeChuan, Chongqing, China; 4 Department of Cancer Center, The Second Affiliated Hospital of Chongqing Medical University, Chongqing, China; 5 Department of Hepatobiliary Surgery, The Second Affiliated Hospital of Chongqing Medical University, Chongqing, China

**Keywords:** biliary tract malignancies, lymph node, machine learning, prognosis, random forest (RF) model

## Abstract

**Objective:**

This study aimed to construct a new machine learning model based on multiple risk factors to predict the prognosis of patients with biliary tract malignancies after surgery.

**Materials and methods:**

This study included 6,951 patients in the SEER database from 2004 to 2015, and 245 patients in our medical center from 2015 to 2022. The patients in the SEER database were divided into a training set and a test set in a 7:3 ratio. The data from this research center serves as the external validation set. The clinical outcome was overall survival (OS). Univariate and multivariate Cox regression analyses were used in the training set to screen out independent prognostic factors. Then, multiple machine learning models were constructed to predict the survival rates of patients at 1 year, 3 years and 5 years. The performance of the model was evaluated using the area under the curve (AUC), calibration curves, and the decision curve analysis (DCA). SHAP analysis was used to identify features with greater contributions to model prediction. Subgroup analysis and survival curves were used to evaluate the benefits of chemotherapy for patients with positive lymph nodes.

**Results:**

Age, sex, marital status, tumor site, tumor differentiation, AJCC stage, regional nodes examined, regional nodes positive, and LN surgery Scope were all independent risk factors affecting the OS of patients with biliary tract malignancies. Among the various models constructed, the random forest (RF) model showed good AUC values across different datasets. Moreover, the calibration curves and decision curve analysis of the RF model in different sets indicate that it has excellent performance. SHAP analysis indicates regional nodes positive, AJCC stage, tumor site, and age play a crucial role in the survival analysis of biliary tract malignancies. Chemotherapy was associated with improved OS among patients with positive lymph nodes in the training and test cohorts.

**Conclusions:**

This study developed a new machine learning model based on multiple risk factors, which can effectively predict the prognosis of patients with biliary tract malignancies after surgery. This model may assist postoperative risk stratification and individualized adjuvant treatment decision-making.

## 1 Introduction

Biliary tract malignancies are a group of aggressive tumors that originate from the epithelial cells of the bile duct or the mucosa of the gallbladder. They are the second most common malignant tumor of the liver and biliary system after hepatocellular carcinoma. In recent years, their global incidence has shown a slow upward trend, attracting widespread attention from the medical community (1–3).

These diseases are characterized by their deep anatomical location, concealed early symptoms, and the lack of effective screening methods, resulting in most patients being diagnosed at an advanced stage and losing the opportunity for radical surgery. Biliary tract malignancies have high heterogeneity and invasiveness (4). Depending on their anatomical origin, they are mainly classified as intrahepatic cholangiocarcinoma, hilar cholangiocarcinoma, distal cholangiocarcinoma, gallbladder cancer, and ampullary periampullary cancer (5, 6). The clinical course of biliary tract malignancies is severe, and the prognosis is extremely poor. For patients with advanced-stage and non-surgical treatment options, traditional chemotherapy has limited effects, and the overall 5-year survival rate is generally low, posing a serious threat to the patient’s life and health. Therefore, it has been recognized as one of the most challenging areas in digestive tract tumors (7, 8).

Therefore, improving survival outcomes in patients with biliary tract malignancies remains an important clinical challenge. At present, many studies have developed prognostic prediction models specifically for patients with biliary tract malignancies (9, 10). For patients with biliary tract malignancies who are eligible for radical treatment, it is of great significance to be able to predict the prognosis through some clinically available variables and to intervene promptly. Machine learning has increasingly been applied as an effective computational approach in biomedical research. Therefore, this study, based on large-scale population data, constructed a new model using machine learning methods to predict the prognosis of patients with biliary tract malignancies after undergoing surgical treatment. The aim was to identify patients with different prognoses and assist in making treatment decisions, thereby helping patients achieve better survival outcomes and quality of life.

## 2 Materials and methods

### 2.1 Data source and description

The data analyzed in this study were sourced from the SEER database and our medical center. The inclusion and exclusion criteria are as follows. Inclusion criteria include: (1) Age > 18 years; (2) Patients with pathologically confirmed biliary tract malignancies (Intrahepatic bile duct, Extrahepatic bile duct, Gallbladder, Ampulla of Vater); (3) Biliary tract malignancy was the only primary malignant tumor; (4) Received radical surgical treatment. Exclusion criteria include: (1) Did not receive radical surgical treatment; (2) Incomplete clinical or follow-up data.

This study retrieved 6,951 patients in the SEER database from 2004 to 2015, and 245 patients in our medical center from 2015 to 2022. The flowchart of this research is shown in **Figure 1**. The patients in the SEER database were divided into a training set and a test set in a 7:3 ratio. The data from this research center serves as the external validation set. The work has been reported in line with the STROCSS criteria (11).

**Figure 1:**
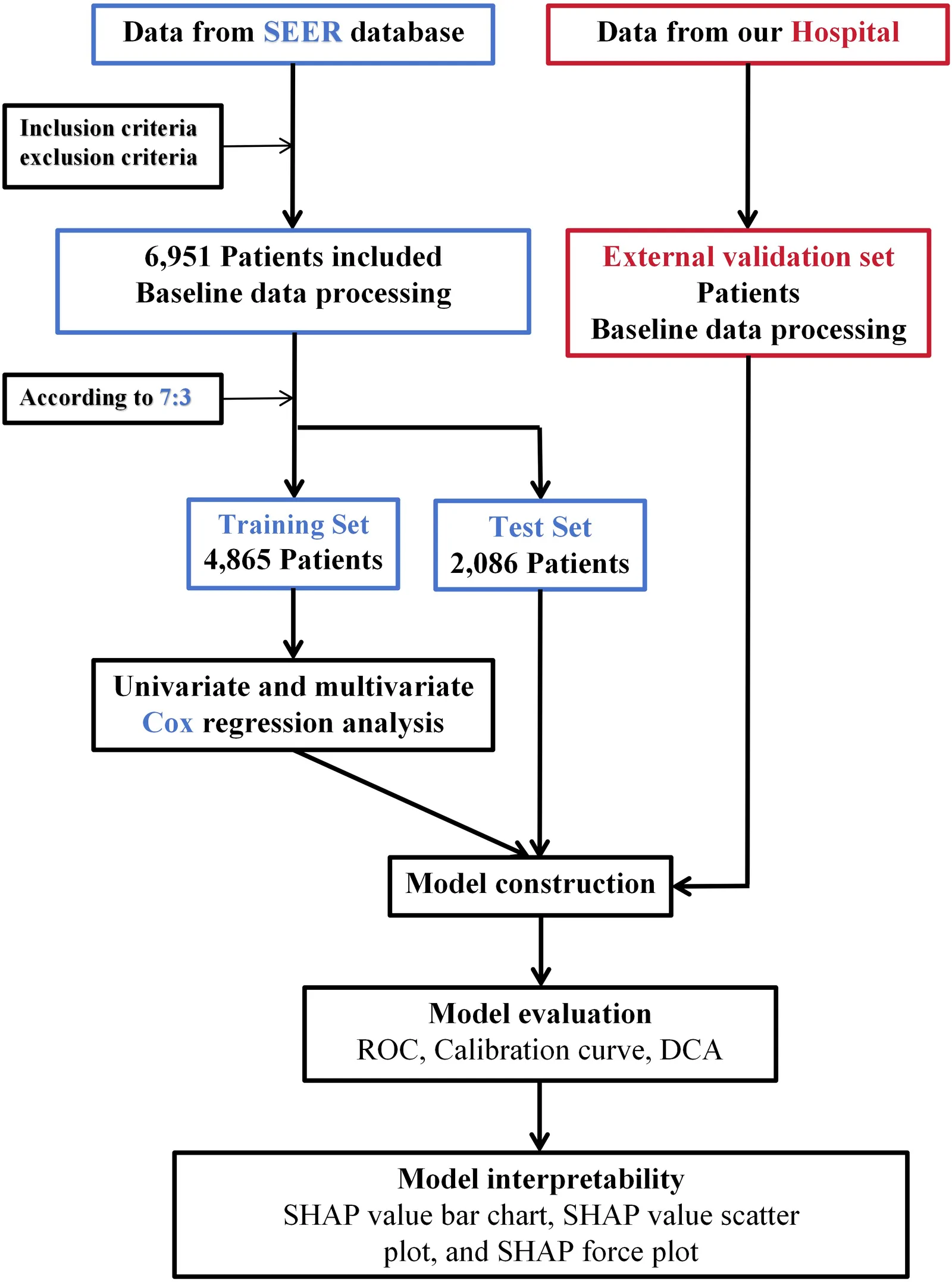
Flowchart of this study.

### 2.2 Variable acquisition and clinical outcomes

The baseline variables included in this study were as follows: age (<65, ≥65), sex (Male, Female), marital status (Unmarried, Married), tumor site (Intrahepatic bile duct, Extrahepatic bile duct, Gallbladder, Ampulla of Vater), Grade Recode (tumor differentiation) (Well, Moderately, Poor, Undifferentiated), AJCC Stage (I, II, III, IV), tumor size, chemotherapy(No, Yes), Regional nodes examined (LN Examined), Regional nodes positive (LN Positive), LN surgery (SUR) Scope, Survival Status, overall survival (OS). “LN SUR Scope” in the SEER database and the data from the single-center study refers to the surgical scope of regional lymph nodes. And “1 to 3” indicates that a portion of the regional lymph nodes have been removed, but it does not meet the standards or recommended range for lymph node dissection for this type of tumor. “4 or more” refers to a lymph node dissection procedure where the scope of dissection is the standard area or even an expanded area, based on the consensus or clinical guidelines for that specific tumor site at that time.

The OS of patients in the SEER database is based on the database. The follow-up deadline for patients at this medical center is June 2025. OS is defined as the time interval from when a patient undergoes radical surgical treatment to their full-fledged death. If death does not occur, it is the time interval from the date of treatment to the end of the follow-up period. The follow-up of patients at this medical center is conducted through the electronic medical record system, outpatient visits, or telephone calls.

### 2.3 Statistical analysis

First, univariate Cox proportional hazards regression analysis was conducted on the clinical and pathological characteristics of patients with biliary tract malignancies in the training set to calculate the hazard ratio (HR) and its 95% confidence interval (CI), and a two-sided P value <0.05 was considered statistically significant. Cox proportional hazards regression was initially performed to identify clinically meaningful independent prognostic factors associated with overall survival. Variables identified as significant in multivariate Cox regression were subsequently incorporated into machine learning models. This approach was adopted to reduce redundant variables, improve model interpretability, and maintain clinical relevance during model construction. On the training set, various machine learning models such as Random Forest (RF), Extreme Gradient Boosting (XGBoost), k-Nearest Neighbors (KNN), Support Vector Machine (SVM), Light Gradient Boosting Machine (LightGBM) and CatBoost were developed to predict the survival rates of patients at 1 year, 3 years and 5 years. The training set was used for hyperparameter tuning and preliminary performance evaluation, and the final performance was verified on the test set and validation set. The parameters of the six machine learning algorithms can be found in **Supplementary File 1**. The model evaluation indicators included the area under the receiver operating characteristic curve (ROC), Brier score, calibration curve based on the Hosmer–Lemeshow test, and decision curve analysis (DCA), which were used to comprehensively evaluate the discrimination, calibration and clinical applicability of the model. The model with the best overall performance was selected. The SHAP (Shapley Additive Explanations) method was used to conduct interpretability analysis, and the feature importance graph was drawn to reveal the key influencing factors and contribution degrees of the best model in predicting survival rates at different time points. Furthermore, we divided the patients into two groups based on whether their lymph nodes were positive after radical surgery. In the training set, test set, and validation set, we respectively explored the potential benefits of chemotherapy. Survival curves were plotted using the Kaplan–Meier method, and the survival differences among the groups were compared through the log-rank test. In addition, we conducted univariate and multivariate Cox regression analyses within different subgroups based on the location of the tumors to identify risk factors. Furthermore, before modeling all the variables and after screening the variables, we calculated the variance inflation factor (VIF) to identify and reduce the interference caused by collinearity, thereby enhancing the stability of the model.

All statistical analyses were conducted in the Python 3.10 environment, SPSS 26.0 and graphpad prism. The Cox regression analysis and survival curve plotting were carried out using SPSS 26.0 and graphpad prism. The time-dependent ROC and Brier score calculations were performed using the scikit-survival library. The machine learning model construction and parameter adjustment were based on the scikit-learn, xgboost, lightgbm, and catboost libraries. The interpretability analysis utilized the shap library, and the plotting was done using the matplotlib and seaborn libraries.

## 3 Results

### 3.1 Baseline characteristics

**Table 1** presents the baseline characteristics of the patients included in this study. The training set, test set and external validation set respectively included 4865, 2086 and 245 patients. The median number of lymph nodes examined during surgery for the training set, test set and external validation set was 8 (3-16), 9 (3-16), and 12 (3-18), respectively. The median number of positive lymph nodes after surgery was 0 (0-2), 1 (0-2), and 0 (0-3), respectively. The median OS of patients in the training set, test set and external validation set were 30 (12-84), 29 (12-83), and 32 (17-53), respectively.

**Table 1:** General characteristics.

Variables		Train set (n=4865)	Test set (n=2086)	Validation set (n=245)
Age	<65	2024 (41.6%)	889 (42.6%)	74 (30.2%)
	≥65	2841 (58.4%)	1197 (57.4%)	171 (69.8%)
Sex	Male	2421 (49.8%)	1080 (51.8%)	159 (64.9%)
	Female	2444 (50.2%)	1006 (48.2%)	86 (35.1%)
Marital Status	Unmarried	1769 (36.4%)	737 (35.3%)	91 (37.1%)
	Married	3096 (63.6%)	1349 (64.7%)	154 (62.9%)
Tumor Site	Intrahepatic bile duct	411 (8.4%)	172 (8.2%)	27 (11.0%)
	Extrahepatic bile duct	1150 (23.6%)	514 (24.6%)	65 (26.5)
	Gallbladder	1334 (27.4%)	499 (23.9%)	27 (11.0%)
	Ampulla of Vater	1970 (40.5%)	901 (43.2%)	126 (51.4%)
Tumor Differentiation	Well	571 (11.7%)	242 (11.6%)	37 (15.1%)
	Moderately	2492 (51.2%)	1071 (51.3%)	143 (58.4%)
	Poor	1724 (35.4%)	736 (35.3%)	62 (25.3%)
	Undifferentiated	78 (1.6%)	37 (1.8%)	3 (1.2%)
AJCC Stage	I	1402 (28.8%)	581 (27.9%)	73 (29.8%)
	II	2165 (44.5%)	955 (45.8%)	111 (45.3%)
	III	962 (19.8%)	412 (19.8%)	61 (24.9%)
	IV	336 (6.9%)	138 (6.6%)	0
Tumor Size (mm)		25 (16-40)	25 (17-39)	27 (21-40)
Chemotherapy	No	2635 (54.2%)	1135 (54.4%)	144 (58.8%)
	Yes	2230 (45.8%)	951 (45.6%)	101 (41.2%)
LN Examined		8 (3-16)	9 (3-16)	12 (3-18)
LN Positive		0 (0-2)	1 (0-2)	0 (0-3)
LN SUR Scope	1 to 3	1503 (30.9%)	594 (28.5%)	73 (29.8%)
	4 or more	3362 (69.1%)	1492 (71.5%)	172 (70.2%)
Survival Status	Alive	1186 (24.4%)	480 (23.0%)	63 (25.7%)
	Dead	3679 (75.6%)	1606 (77.0%)	182 (74.3%)
OS (months)		30 (12-84)	29 (12-83)	32 (17-53)

AJCC, the American Joint Committee on Cancer; LN, lymph nodes; SUR, surgery; OS, overall survival.

### 3.2 Univariate and multivariate Cox regression

The results of univariate Cox regression analysis showed that sex, marital status, tumor site, tumor differentiation, AJCC stage, tumor size, LN examined, LN positive, and LN SUR Scope, as well as age, were all significantly associated with the overall survival (OS) of patients with biliary tract malignancies (p < 0.05, **Table 2**).

**Table 2:** Univariable and multivariable Cox regression analysis of OS in Train set.

Variables		Univariate analysis		Multivariate analysis	
		HR (95% CI)	P value	HR (95% CI)	P value
Age (≥65)		1.515 (1.417,1.620)	**<0.001**	1.526 (1.426,1.634)	**<0.001**
Sex (Female)		1.081 (1.013,1.153)	**0.019**	1.141 (1.065,1.222)	**<0.001**
Marital Status (Married)		0.852 (0.797,0.911)	**<0.001**	0.812 (0.758,0.870)	**<0.001**
Tumor Site	Extrahepatic bile duct	1.195 (1.053,1.356)	**<0.001**	1.322 (1.151,1.518)	**<0.001**
	Gallbladder	1.052 (0.927,1.193)		1.141 (0.998,1.305)	
	Ampulla of Vater	0.787 (0.697,0.890)		0.891 (0.777,1.022)	
Tumor Differentiation	Moderately	1.179 (1.057,1.315)	**<0.001**	1.090 (0.976,1.217)	**<0.001**
	Poor	1.681 (1.503,1.881)		1.326 (1.183,1.487)	
	Undifferentiated	2.381 (1.845,3.071)		1.443 (1.115,1.867)	
AJCC Stage	II	2.085 (1.918,2.267)	**<0.001**	1.969 (1.805,2.148)	**<0.001**
	III	2.380 (2.159,2.625)		2.418 (2.174,2.691)	
	IV	4.479 (3.923,5.114)		3.542 (3.082,4.070)	
Tumor Size		1.001 (1.000,1.001)	**0.021**	1.000 (0.999,1.001)	0.932
Chemotherapy (Yes)		0.973 (0.912,1.038)	0.410		
LN Examined		0.992 (0.988,0.995)	**<0.001**	0.991 (0.987,0.996)	**<0.001**
LN Positive		1.082 (1.072,1.092)	**<0.001**	1.065 (1.053,1.077)	**<0.001**
LN SUR Scope (4 or more)		0.797 (0.744,0.854)	**<0.001**	0.811 (0.740,0.888)	**<0.001**

OS, overall survival; HR, hazard ratio; CI, confidence interval; AJCC, the American Joint Committee on Cancer; LN, lymph nodes; SUR, surgery. The bold P-value indicates a statistically significant difference.

To control for potential confounding factors, multivariate Cox regression analysis was further conducted to identify the independent prognostic factors affecting OS. The results indicated that sex, marital status, tumor site, tumor differentiation, AJCC stage, LN examined, LN positive, and LN SUR Scope, as well as age, were all independent risk factors affecting the OS of patients with biliary tract malignancies (p < 0.05, **Table 2**). The risk factors identified through univariate and multivariate Cox regression analysis based on the tumor site were listed in **Supplementary File 2**. Except for intrahepatic cholangiocarcinoma with a smaller sample size, the analyses of the other subgroups all supported the risk factors included in this study. This provides a basis for combining analysis of key predictive factors.

To assess multicollinearity, we calculated the VIF both before and after feature selection. The results showed that the VIF of all variables was below 5, indicating that there was no significant collinearity problem in the model (**Supplementary File 3**).

### 3.3 Model establishment and evaluation

The clinical characteristics with statistical significance (P < 0.05) in the multivariate Cox regression analysis were selected and included in the machine learning modeling process to retain the key information related to the survival outcome to the greatest extent. Based on this, six different machine learning prediction models were constructed to evaluate the survival rates of patients with biliary tract malignancies at 1 year, 3 years, and 5 years. During the model training process, ten-fold cross-validation iteration and systematic hyperparameter optimization were adopted to obtain stable and highly generalized model parameter combinations. To comprehensively evaluate the model performance, the AUC values, Brier scores, and confusion matrices of the six models on the training set, validation set, and test set were compared in multiple dimensions (**Figures 2**, **3**). The results showed that the random forest (RF) model performed outstandingly in all performance indicators, especially in terms of generalization ability and prediction stability. Specifically, the AUC of the RF model in predicting the survival rate of patients with biliary tract malignancies was as follows: 1 year (Training set AUC = 0.758, Testing set AUC = 0.707, Validation set AUC = 0.796), 3 years (Training set AUC = 0.797, Testing set AUC = 0.749, Validation set AUC = 0.870), and 5 years (Training set AUC = 0.850, Testing set AUC = 0.740, Validation set AUC = 0.886) (**Figures 2**, **3**).

**Figure 2:**
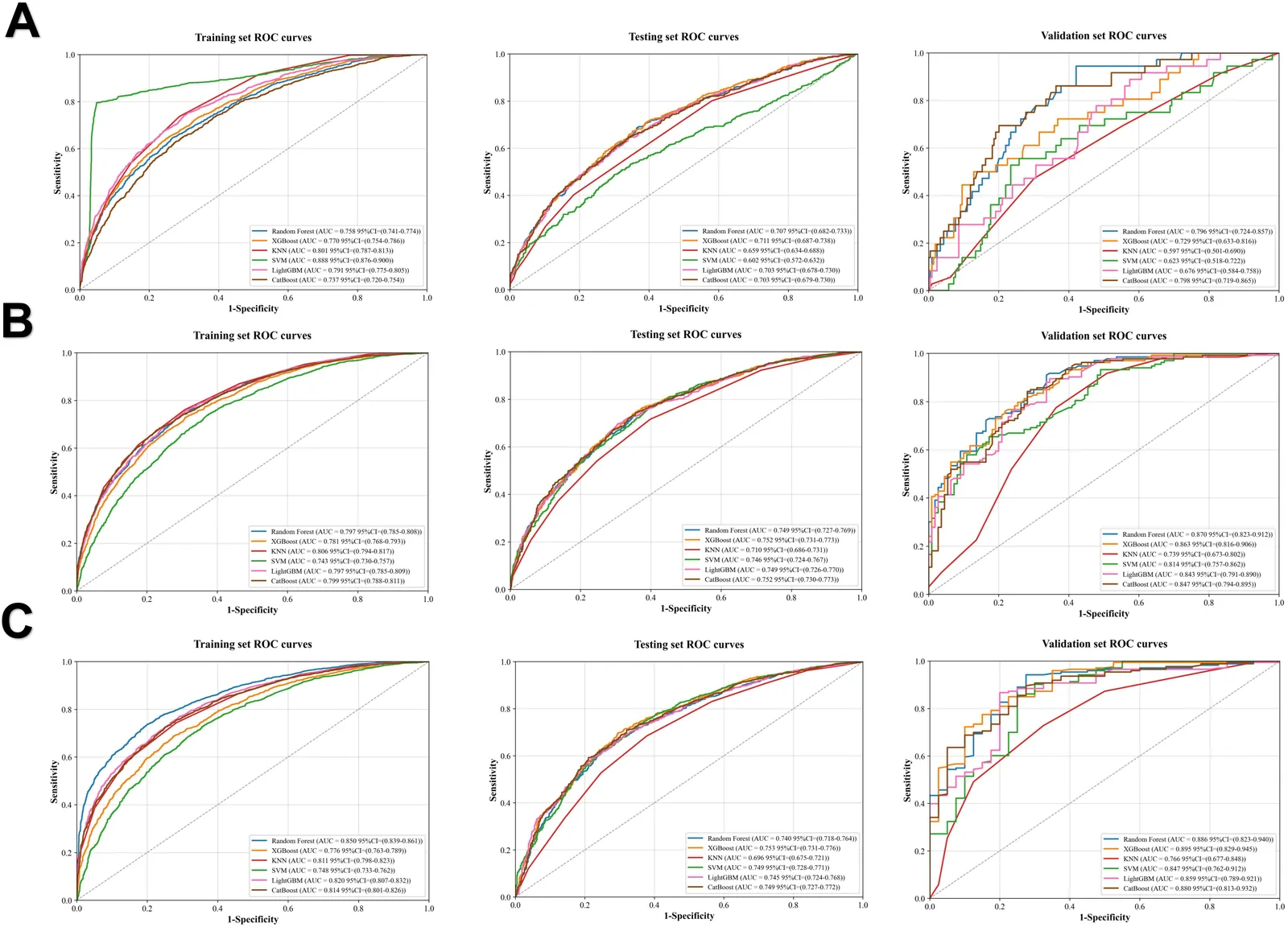
ROC curves of different machine learning models on the training set, test set and validation set. **(A)** the ROC curves of the 1-year prognosis model training set, test set and validation set); **(B)** the ROC curves of the 3-year prognosis model (training set, test set and validation set); **(C)** the ROC curves of the 5-year prognosis model (training set, test set and validation set).

**Figure 3:**
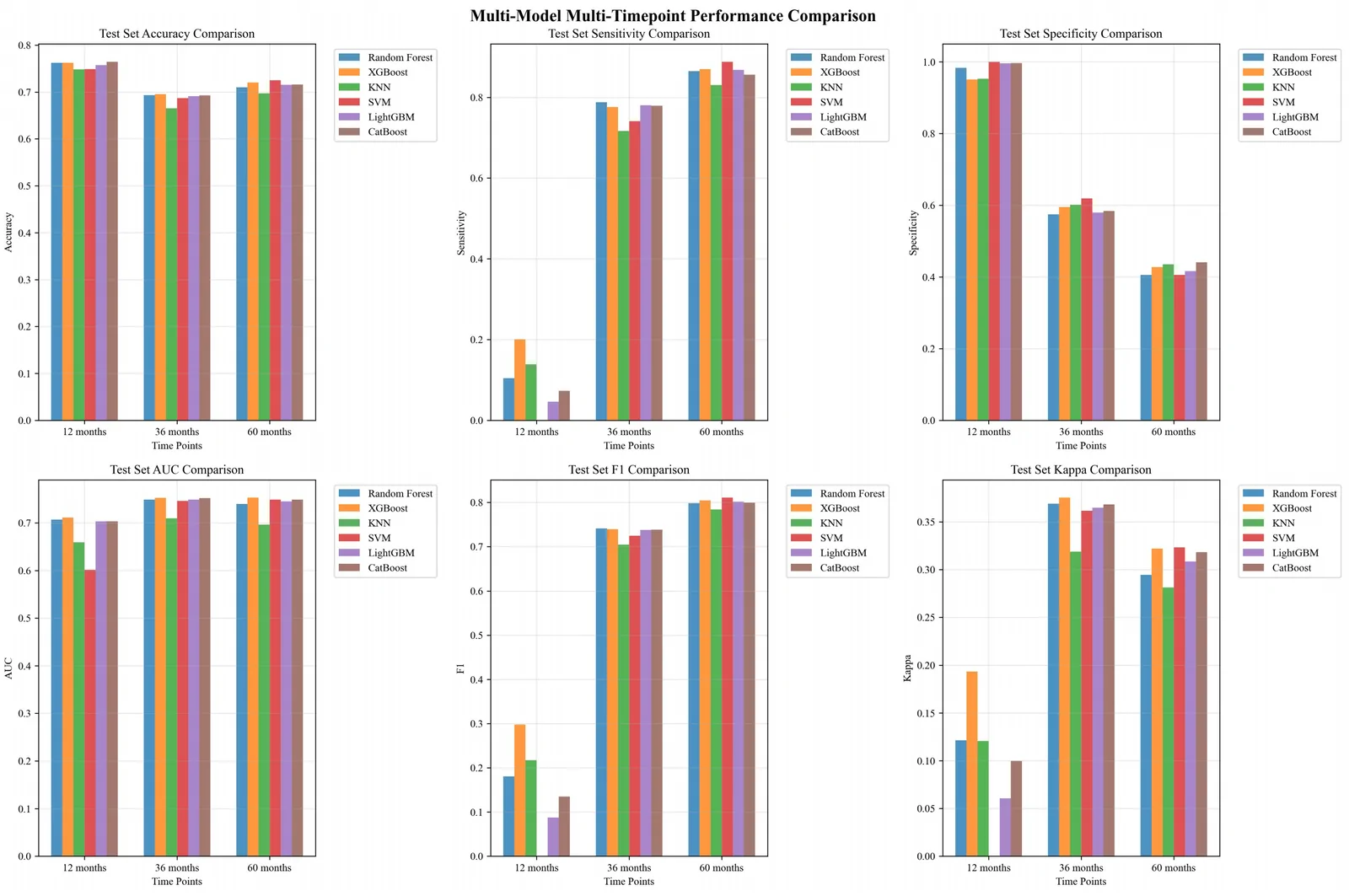
Multi-model multi-timepoint performance comparison in accuracy, sensitivity, specificity, AUC, F1, kappa.

In addition, the calibration curves drawn in the training set, test set and validation set showed that the prediction probabilities of the RF model at each time point were consistent with the actual observed survival rates (**Figure 4**). The decision curve analysis also indicated that in a wide range of threshold probabilities, the RF model could provide higher net clinical benefits, superior to other control models (**Figure 5**). The confusion matrix analysis further supported the generalization ability of the RF model, which showed a high proportion of true positives and true negatives in the test and validation cohorts, with a relatively small number of misclassifications (**Figure 6**; **Supplementary Figures 1**, **2**). In contrast, the other models had a higher false positive rate in the test and validation cohorts, which was consistent with their relatively lower generalization performance. Based on the above results, the RF model demonstrated favorable predictive performance across different datasets. However, the interpretation of long-term survival prediction in the external validation cohort should be considered cautiously because of the limited follow-up duration and potential influence of right-censoring.

**Figure 4:**
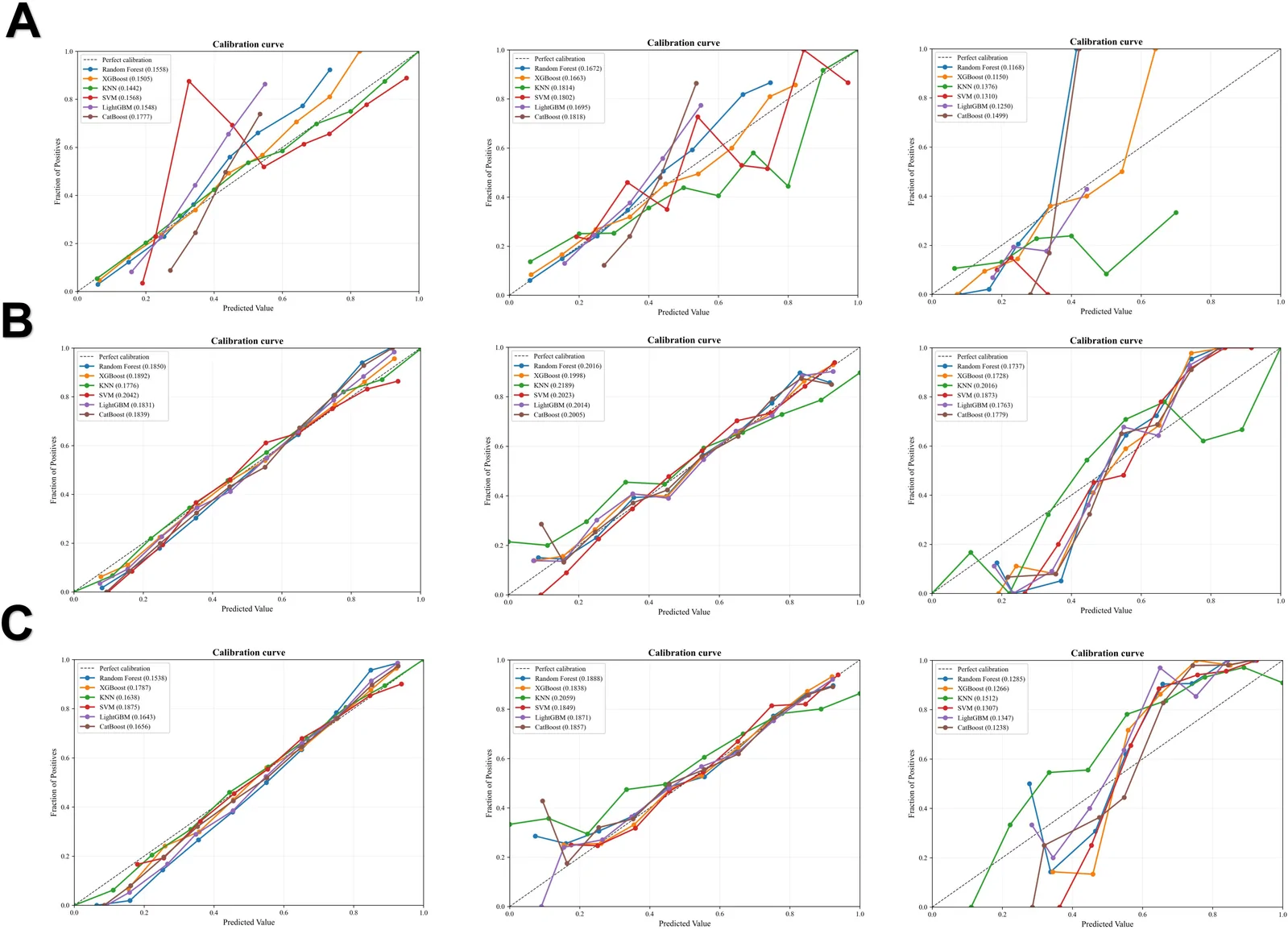
Calibration curves of different machine learning models on the training set, test set and validation set. **(A)** the ROC curves of the 1-year prognosis model training set, test set and validation set); **(B)** the ROC curves of the 3-year prognosis model (training set, test set and validation set); **(C)** the ROC curves of the 5-year prognosis model (training set, test set and validation set).

**Figure 5:**
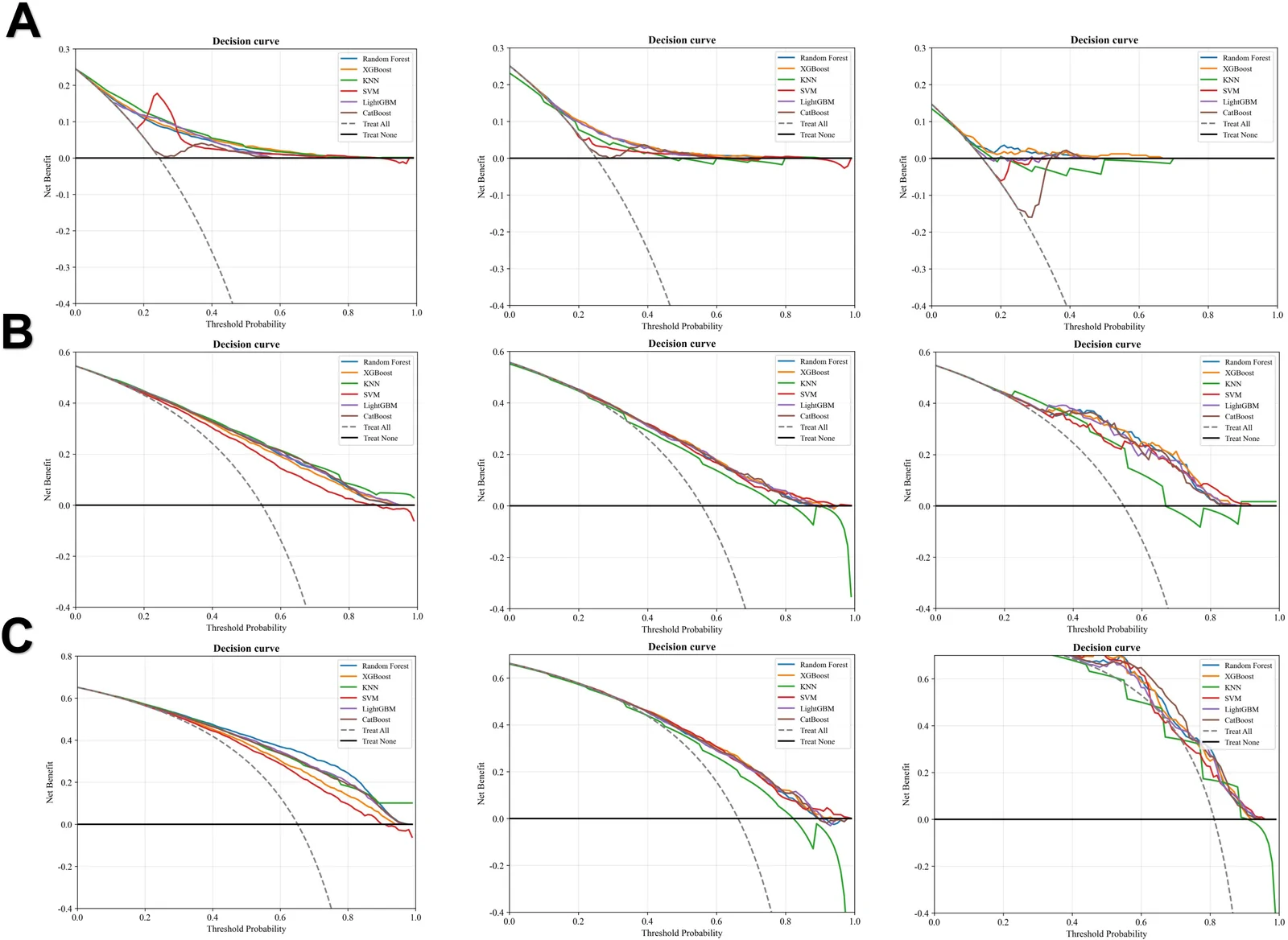
Decision curve analysis of different machine learning models on the training set, test set and validation set. **(A)** the ROC curves of the 1-year prognosis model training set, test set and validation set); **(B)** the ROC curves of the 3-year prognosis model (training set, test set and validation set); **(C)** the ROC curves of the 5-year prognosis model (training set, test set and validation set).

**Figure 6:**
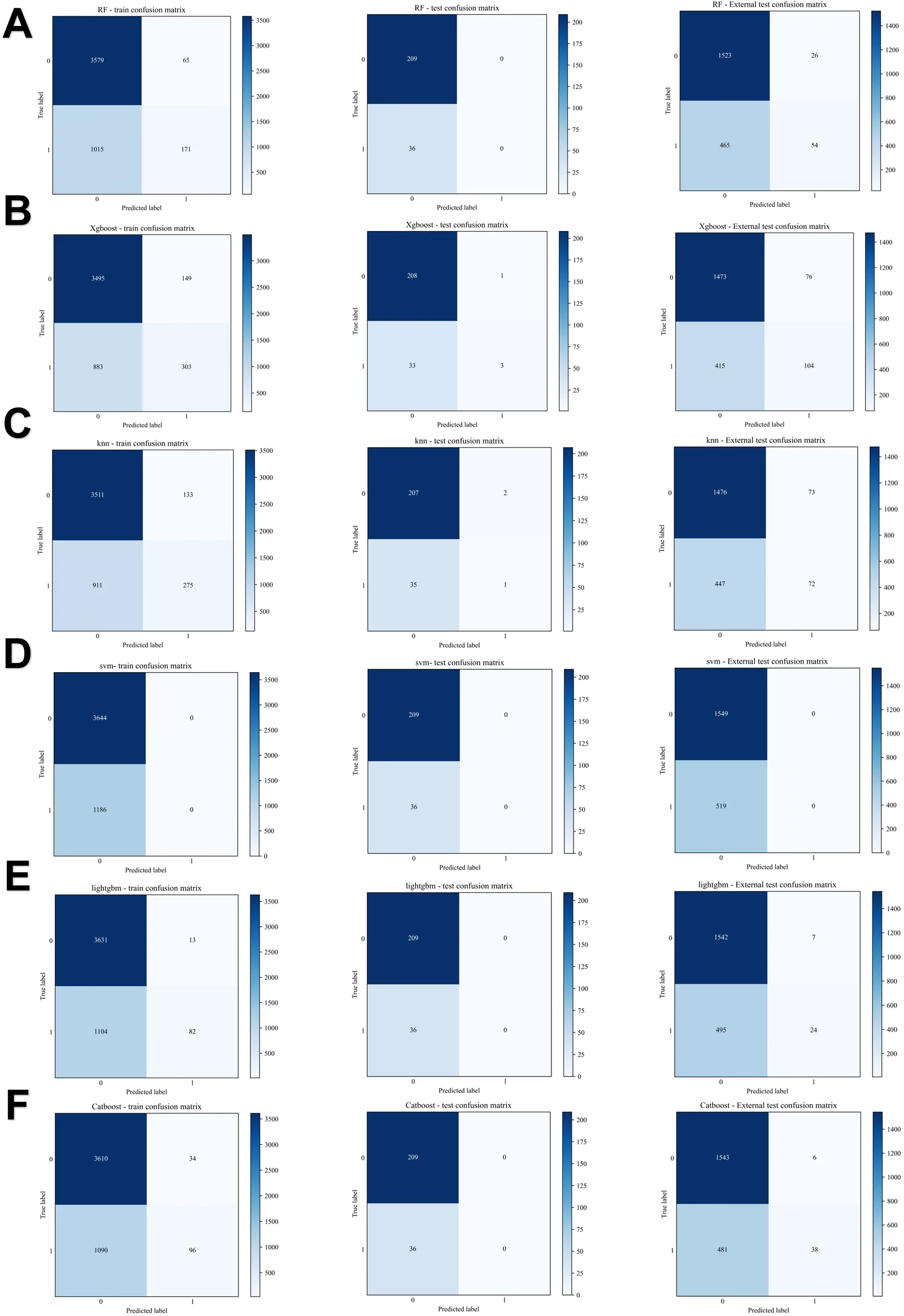
Confusion matrices of different models in predicting 1-year prognosis. **(A)** Random Forest; **(B)** XGBoost; **(C)** KNN; **(D)** SVM; **(E)** LightGBM; **(F)** CatBoost.

### 3.4 Model explanation and SHAP analysis

**Figure 7** presents a bar chart of SHAP values for the survival analysis of biliary tract malignancies, used to quantify and visualize the contribution of each feature to the model’s prediction. The SHAP value indicates the degree of influence of each feature on the final prediction result under different feature combinations. A higher SHAP value implies a greater impact of the feature on the model’s prediction. **Figure 7A** is a combined display of the SHAP bar chart and the swarm plot, which can visually present the ranking of feature importance and their specific impacts on the prediction result in different value ranges. The SHAP values are color-coded (blue represents a lower feature value, and red represents a higher feature value), revealing the relationship between feature values and the prediction results. From **Figure 7A**, it can be seen that regional nodes positive, AJCC stage, tumor site, and age are the four most important features in the model’s prediction, with their contributions higher than those of other features. In contrast, features such as tumor differentiation, regional nodes examined, marital status, LN Sur Scope, and sex have a smaller impact. This indicates that regional nodes positive, AJCC stage, tumor site, and age play a crucial role in the one-year survival analysis of biliary tract malignancies, while the influence of other factors is weaker. The analysis of this combined graph not only reveals the key variables relied upon by the model but also provides potential reference value for clinical risk stratification and individualized prognosis assessment.

**Figure 7:**
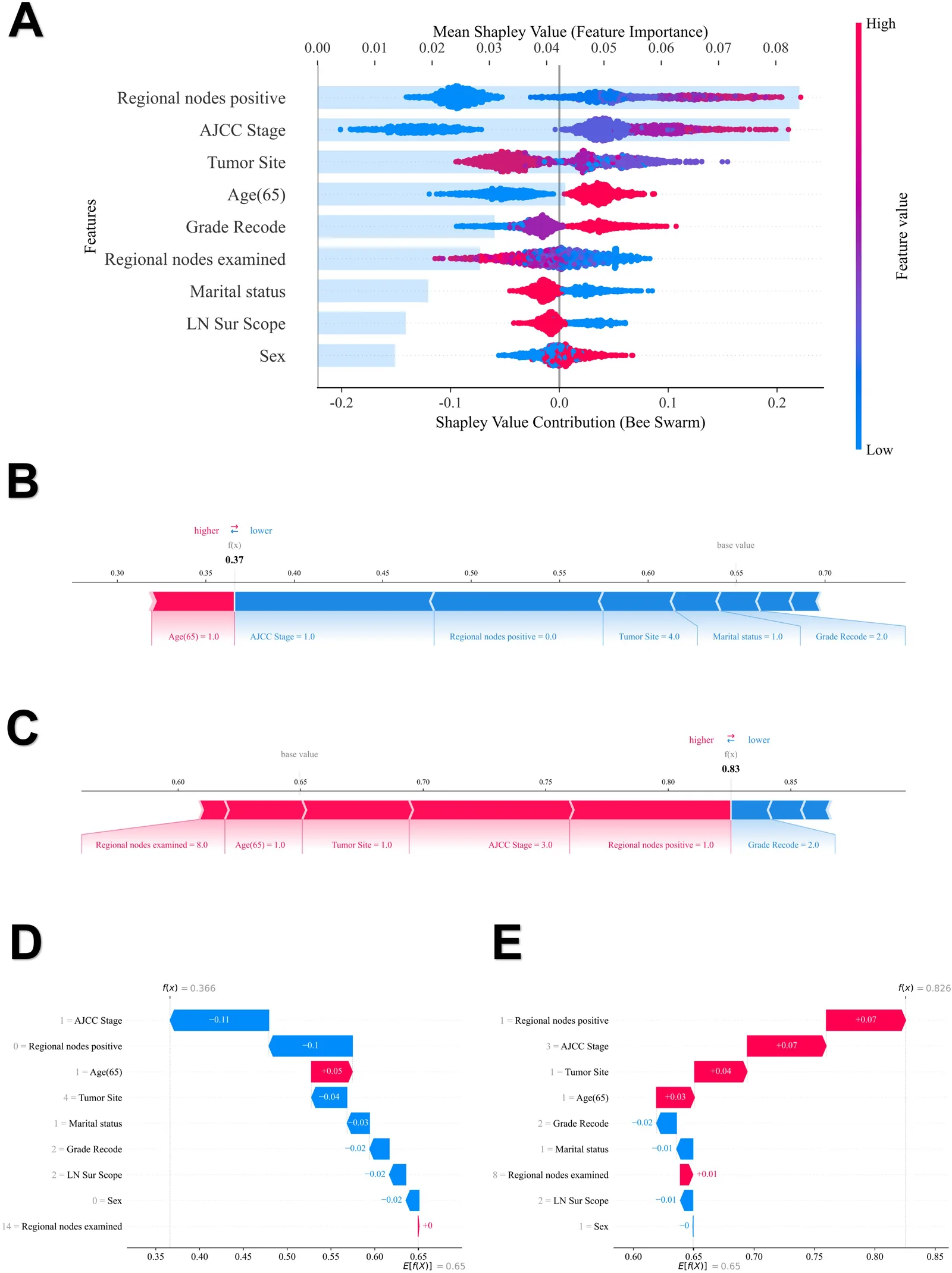
SHAP analysis for quantifying and visualizing the contribution of each feature to the model in predicting 1-year prognosis. **(A)** The combination of SHAP bar chart and bee colony chart; **(B, C)** Force plots; **(D, E)** Waterfall plots.

**Figures 7B, C** are force plots, used to illustrate how each feature works together on the basis of the model’s baseline prediction value (base value) to form the final prediction value f(x). The blue arrows represent features that lower the prediction value, while the red arrows represent features that increase the prediction value. **Figures 7D, E** are waterfall plots, showing the contribution of different features to the model’s prediction output (SHAP value) for a particular patient. Each bar represents a feature, where blue indicates a negative contribution to the prediction result (lowering the risk), and red indicates a positive contribution (increasing the risk). **Figures 7B, D** correspond to the same biliary tract malignancies survival patient. Among them, AJCC=I, regional nodes positive = No, tumor site = Ampulla of Vater, Marital status=Married, and tumor differentiation = Moderately are the main risk reduction factors, significantly lowering the model’s prediction value, suggesting that the patient’s mortality risk is lower. At the same time, Age ≥65 years was a risk-increasing factor. The final prediction value of this patient is 0.366, indicating a lower mortality risk. **Figures 7C, E** correspond to another biliary tract malignancies death patient. Their tumor differentiation = Moderately lead to a significant increase in the model’s prediction value. And regional nodes examined =8, Age≥65, tumor site= Intrahepatic bile duct, AJCC=III, and regional nodes positive=Yes are risk increase factors. The final prediction value of this patient is 0.826, suggesting an extremely high mortality risk.

In addition, the 1-year and 3-year SHAP analysis results of biliary tract malignancies have been presented in the **Supplementary File** for further reference (**Supplementary Figures 3**, **4**).

### 3.5 The benefits of chemotherapy for patients

The patients in the training set, test set and validation set were divided into subgroups based on whether their postoperative lymph nodes were positive. This was done to explore the benefits of chemotherapy for the patients. As shown in **Figures 8A, B**, in both the training set and the test set, among the subgroup of patients with negative lymph nodes, chemotherapy did not improve the OS of the patients. In the subgroup with positive lymph nodes, patients who received chemotherapy had a significantly longer OS compared to those who did not receive chemotherapy (P < 0.05). As shown in **Figure 8C**, in the validation set, in the subgroup with negative lymph nodes, patients who received chemotherapy had a longer OS compared to those who did not receive chemotherapy (P < 0.05). In the subgroup with positive lymph nodes, chemotherapy did not improve the OS of the patients. The reason for this situation might be that the number of patients in the validation set is relatively small, which introduces a certain degree of randomness.

**Figure 8:**
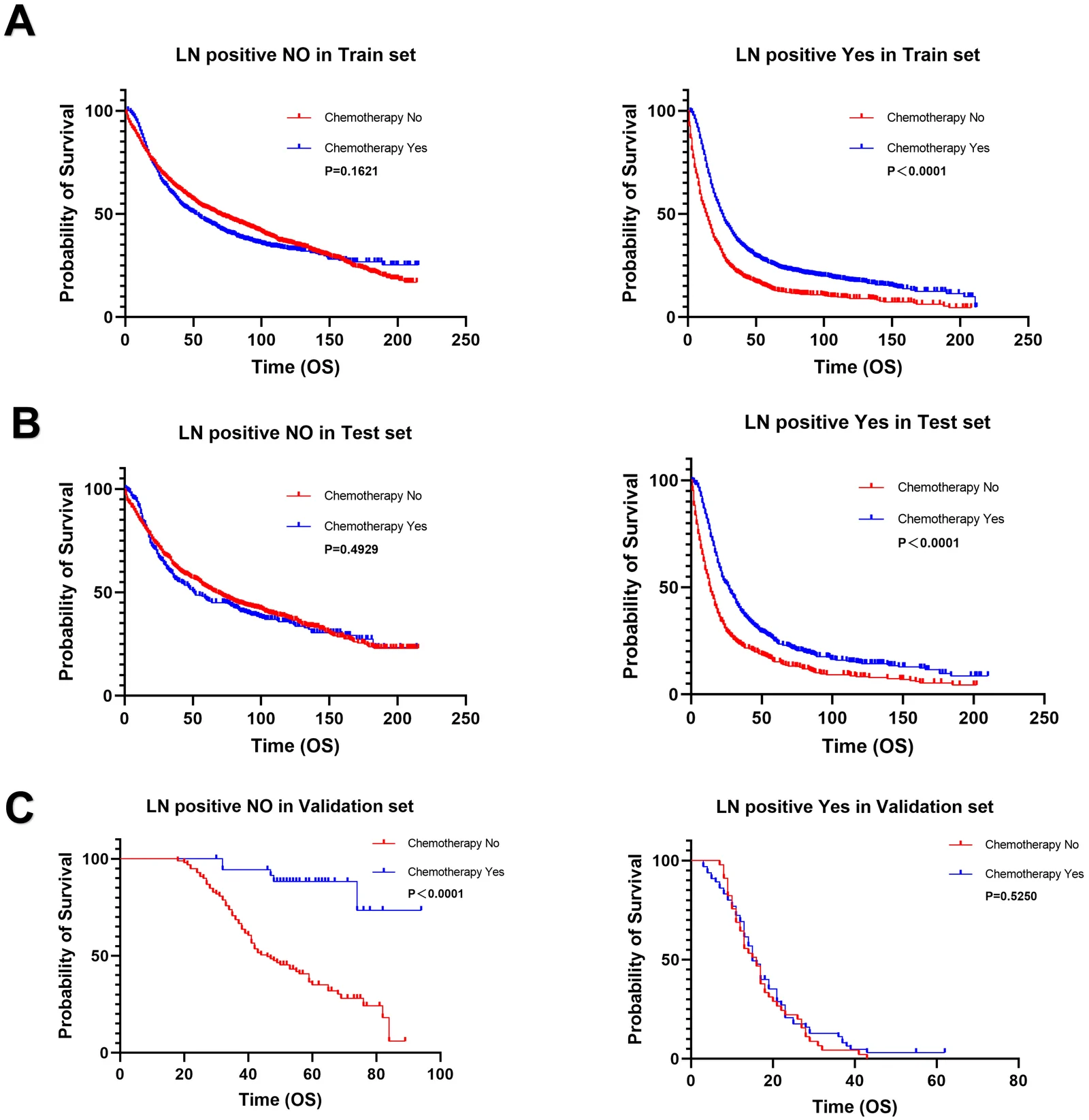
Survival analysis of patients with biliary tract malignancies with positive lymph nodes after surgery regarding their benefit from chemotherapy. **(A)** Training set; **(B)** Test set; **(C)** Validation set.

## 4 Discussion

Biliary tract malignancies are a group of diseases that are difficult to diagnose and has a poor prognosis. They represent a major challenge for current liver and biliary surgery as well as oncology (12, 13). A deeper understanding of their biological characteristics, the promotion of the development of early diagnosis technologies, and the exploration of individualized precision treatment based on molecular stratification are the core and future direction for improving patient prognosis and enhancing quality of life. Many current studies are exploring how to predict and improve the prognosis of patients with biliary tract malignancies. The traditional AJCC/TNM staging system mainly relies on anatomical scope, although it is important, it cannot fully capture the complexity of patients (such as tumor biological behavior, genomic characteristics) and treatment (14). However, machine learning models can integrate multi-dimensional data (such as clinical pathology, radiomics, genomics, surgical details, etc.), providing more precise and personalized predictions of recurrence risk and survival probability, potentially improving upon the limitations of traditional staging systems (15, 16). Compared with conventional staging systems, machine learning approaches may integrate multiple clinical and pathological variables simultaneously and provide individualized survival probability estimation.

Therefore, in this study, a new prognostic prediction model based on multiple risk factors was constructed based on a large dataset of patients with biliary tract malignancies using machine learning methods. Univariate and multivariate Cox proportional hazards regression analyses were employed to identify independent prognostic factors in the training set. After constructing various machine learning models in the training set, the evaluation and validation of the models were carried out in the test set and the validation set. It was ultimately found that the RF model had a significant advantage in predicting the prognosis of patients with biliary tract malignancies. The AUC values of the RF model in predicting the survival rate of patients with biliary tract malignancies were as follows: 1 year (Training set AUC = 0.758, Testing set AUC = 0.707, Validation set AUC = 0.796), 3 years (Training set AUC = 0.797, Testing set AUC = 0.749, Validation set AUC = 0.870), and 5 years (Training set AUC = 0.850, Testing set AUC = 0.740, Validation set AUC = 0.886). The calibration curves showed that the predicted probabilities of the RF model at each time point were highly consistent with the actual observed survival rates. Decision curve analysis also indicated that within a wide range of threshold probability values, the RF model could provide higher net clinical benefits, outperforming other control models. Therefore, in this study, the RF model showed the best overall performance among the evaluated models for predicting the long-term survival rate of patients with biliary tract malignancies and had potential clinical application value. Although tumor site was included as a predictive variable and subgroup analyses were performed, the current model was developed using a pooled cohort of biliary tract malignancies. Given the biological heterogeneity among intrahepatic cholangiocarcinoma, extrahepatic cholangiocarcinoma, gallbladder cancer, and ampullary carcinoma, subtype-specific models might provide additional prognostic value in future studies. Therefore, the applicability of the current model to individual tumor subtypes should be interpreted cautiously.

Through SHAP analysis, this study also found that regional nodes positive, AJCC stage, tumor site, and age were the four most important features in the model prediction, with their contributions being higher than those of other features. SHAP analysis provided additional interpretability beyond conventional prognostic models. The consistent importance of lymph node status, AJCC stage, tumor location, and age indicates that these variables contribute substantially to postoperative survival prediction. Clinically, lymph node positivity reflects tumor dissemination potential, while AJCC stage represents anatomical tumor burden and invasiveness. Age may indirectly reflect physiological reserve, treatment tolerance, and postoperative recovery ability. The extent of regional lymph node dissection, the total number of lymph nodes detected, and the number of positive lymph nodes all have an extremely close and well-established relationship with the prognosis of patients with biliary tract malignancies. The number of positive lymph nodes is one of the powerful prognostic indicators for patients with biliary tract malignancies. The number of positive lymph nodes directly reflects the tumor’s invasive and metastatic capabilities. A greater number of positive lymph nodes may indicate a larger tumor burden, and extensive lymph node metastasis implies that tumor cells are likely to have entered the circulatory system, becoming the “seeds” for distant metastasis (such as liver, lung, and peritoneal metastasis) after surgery. The more positive lymph nodes there are, the worse the prognosis may be for the patient. The number of lymph nodes removed during regional lymph node dissection is related to a more accurate staging and possible survival benefits. Some retrospective studies have shown that the greater the total number of lymph nodes removed, the trend of improved survival period for patients exists. This may be because the dissection of lymph nodes clears some micrometastatic lesions, reducing the tumor burden in the patient’s body (17–20).

The AJCC stage is one of the most fundamental and important indicators for predicting the prognosis of patients with biliary tract malignancies. It describes the extent of local infiltration and anatomical invasion of the tumor, and is closely related to the patient’s prognosis. The higher the AJCC stage, the more likely it is that the patient’s prognosis will be worse. The higher the AJCC stage, the larger the tumor volume, the wider the invasion range, and the closer the relationship with surrounding important blood vessels and organs. This makes it difficult for surgeons to obtain a “safe and clean” surgical margin. The risks of R1 (microscopic residue) or R2 (macroscopic residue) resection increase sharply. Residual tumor cells are the root cause of local recurrence after surgery. Moreover, performing surgery on high AJCC-stage tumors often requires more extensive tissue resection, more complex vascular reconstruction, longer operation time, and more bleeding. This directly leads to an increase in the risk of postoperative complications (such as infection, bile leakage, liver failure), which in turn affect the patient’s recovery and the progress of subsequent adjuvant treatment, indirectly resulting in a poor prognosis (21–23).

There are differences in the prognosis of patients with various types of biliary tract malignancies. Gallbladder cancer is usually the most unfavorable type of biliary tract malignancies. This might be because the early symptoms of gallbladder cancer are subtle and difficult to detect, and the diagnosis is usually made at an advanced stage. The lymphatic vessels in the gallbladder are abundant, and early-stage gallbladder cancer is prone to extensive lymphatic metastasis. Moreover, the gallbladder is closely attached to the liver and is prone to invading the liver parenchyma, increasing the surgical difficulty (24, 25). However, the prognosis of ampullary tumors is often relatively better, which may be related to their anatomical location. Their anatomical location is unique, and early patients will develop obstructive jaundice, which is easily noticed by both patients and doctors. Compared with other patients with biliary tract malignancies, the invasiveness of ampullary tumors is relatively lower. And ampullary cancer is also relatively sensitive to postoperative chemotherapy. These factors can all improve the prognosis of patients (26, 27).

A large number of clinical retrospective studies and database analyses have shown that as people age, especially beyond the age of 65 or 70, the overall survival rate of patients with malignant tumors tends to decline. Older patients may attribute the symptoms related to the tumor to old age or other diseases due to coexisting chronic conditions, resulting in delayed medical consultation. In addition, elderly patients have poorer cardiac and pulmonary functions, as well as liver and kidney functions. The risk of postoperative complications (such as infection, anastomotic leakage) increases, which leads to some patients being unable to undergo radical surgery or only being able to undergo palliative surgery. Even if they undergo surgery, the perioperative mortality rate is also relatively high. Moreover, some current studies suggest that tumors in elderly patients may exhibit more malignant biological characteristics (28–30). Although feature interaction effects, such as the potential interaction between age and AJCC stage, may influence survival prediction, the current analysis mainly focused on the contribution of individual variables. Future studies incorporating more comprehensive interaction analyses may further improve understanding of complex prognostic relationships.

These variables play a significant role in predicting the prognosis of patients with biliary tract malignancies. In the future, they may become important clinical indicators that need to be focused on for patients with biliary tract malignancies.

In this study, it was also found that chemotherapy was associated with different survival outcomes of patients with positive lymph nodes after surgery. Although chemotherapy benefit results in the validation set contradict the training/test sets, it is possible that the following reasons contributed to this: Firstly, the overall sample size of the external validation set is relatively small, with limited sample size for the subgroup of patients with positive lymph nodes and those who received chemotherapy. This small sample size significantly reduces the efficacy of statistical tests and increases the risk of type II error (false negative). Even if the true benefit effect exists, it may not be statistically evident due to insufficient event numbers. Secondly, the chemotherapy information recorded in the SEER database is rather limited, lacking specific drug regimens, treatment cycles, and dosage. While our single-center validation set can confirm the implementation of chemotherapy, its treatment regimens may vary significantly over time and depending on individual patient conditions. This unmeasured treatment variability may dilute the overall effect observed in this study. Moreover, although the model controlled for major factors such as age and stage, key clinical variables that influence chemotherapy decisions and tolerance (such as the patient’s comorbidities, liver and kidney function status, and postoperative complications) may have recording differences between the database and the single-center medical records. The external validation center’s decision to administer chemotherapy to patients may be subject to patient selection bias due to potential confounding factors. These factors may collectively contribute to the observed discrepancy. In such cases, patients with positive lymph nodes after surgery may potentially benefit from chemotherapy. However, this observation should be interpreted cautiously because the external validation cohort did not demonstrate the same statistically significant benefit. These findings should be considered exploratory rather than definitive evidence of chemotherapy benefit. Therefore, preoperative neoadjuvant treatment is necessary for patients with suspicious lymph node invasion. Postoperative chemotherapy can also be combined with surgical treatment to reduce the possibility of postoperative recurrence. Moreover, regular chemotherapy intervention can also eliminate some residual tumor cells in the circulatory system, thereby improving the prognosis of patients. These findings provide preliminary evidence regarding potential chemotherapy benefit patterns. However, further prospective and multicenter studies are required before definitive treatment recommendations can be established.

Overall, this study, basing on some common baseline data and clinical pathological data of a large sample of patients with biliary tract malignancies, especially the data relating to lymph nodes, constructed a new model for predicting the postoperative prognosis of patients with biliary tract malignancies through machine learning methods. After being tested and verified, this model has been proven to have good predictive capabilities and feasibility. Compared with other studies, this research constructed a more superior RF model with a large sample size to predict the prognosis of patients with biliary tract malignancies. And by predicting the individualized survival probabilities of patients at different time points, it provides a more direct basis for clinical decision-making. During the process of incorporating risk factors, this study not only included positive lymph nodes, but also integrated key variables such as “regional nodes examined” and “ LN surgery Scope “, providing a more comprehensive assessment of the impact of lymph node burden and intraoperative lymph node dissection on prognosis. Meanwhile, this study also incorporated SHAP analysis to explore the specific contribution direction and magnitude of each risk factor to the prediction of individual patients. This significantly enhances the transparency and clinical credibility of the model, making the prediction results of this study more acceptable and applicable in practice. Furthermore, given the significance of the status of positive lymph nodes, this study conducted subgroup analyses and clearly verified that chemotherapy provided significant survival benefits for patients with positive lymph nodes. This makes the model not only a predictive tool, but also directly provides high-level evidence support for preoperative and postoperative adjuvant treatment decisions. This is the advantage and innovation of the model constructed in this research. Through these models, in clinical practice, empirical medicine can be transformed into data-driven precision medicine. These models can convert complex clinical data into predictive information with clinical action guidance significance, thereby providing individualized and quantitative survival probability predictions for patients with postoperative biliary tract malignancies. The core clinical application lies in assisting in postoperative adjuvant treatment decisions, helping to identify high-risk patients and suggesting potential benefits of chemotherapy from a big data perspective. It can also be used for preoperative doctor-patient communication and treatment planning. In the future, by developing convenient clinical tools and integrating them into the diagnosis and treatment process, this model is expected to become a powerful assistant in improving the stratification management and individualized treatment of patients with biliary tract malignancies, helping them achieve better survival outcomes and quality of life, and ultimately promoting the overall progress of disease diagnosis and treatment.

However, this study still has certain limitations. Firstly, the data for this study were obtained from the SEER database and our medical center. It is a retrospective study and cannot replace a prospective study to prove the strong correlation between prognostic factors and outcomes. Furthermore, the variables in this database are limited, for instance, specific information regarding the surgery. And the absence of some core data may lead to interference from potential confounding variables. Several clinically important variables, including resection margin status (R0/R1/R2), CA19–9 level, perineural invasion, vascular or lymphovascular invasion, and detailed chemotherapy regimens, were unavailable or inconsistently recorded in the current datasets. The absence of these variables may limit the comprehensive assessment of tumor biology and treatment response. Future studies incorporating more detailed pathological and molecular information are warranted. Although Cox regression-based feature screening allowed incorporation of clinically meaningful predictors, other machine learning-based feature selection approaches were not explored. Future studies may compare different feature selection strategies to further optimize model construction. Then, although this study has undergone external validation, there are two limitations of the external validation set: a small sample size (n = 245) and an uneven distribution of tumor locations. These clearly restrict the generalization ability of the final evaluation model. The relatively small sample size in the external validation set will reduce the power of the statistical test and increase the uncertainty of the results of this study. In particular, the external validation cohort included patients diagnosed between 2015 and 2022, and some patients had insufficient follow-up duration for complete 5-year survival assessment. Therefore, the high 5-year AUC observed in the external validation cohort should be interpreted cautiously, as right-censoring and limited event numbers may affect the estimation of long-term predictive performance. Despite this limitation, the model still demonstrated excellent discrimination ability on the small sample external validation set, which is a positive sign. The uneven distribution of tumor sites in the external validation set has an impact on the performance bias of the model. For this study, the external dataset verified the model’s usability in the actual case combinations faced by this center, but failed to fully demonstrate its universal applicability to all possible case combinations. Therefore, the current external validation results support the model’s initial generalization ability, but they are far from being conclusive. Future studies may require multi-center, prospective research, or even basic research to validate the results of this study. Finally, in this study, the tumor location was included as a variable in the screening of risk factors. Therefore, the study analyzed different subtypes of biliary tract malignancies. Although this study conducted univariate and multivariate Cox regression analyses based on tumor location to subdivide the data and clearly demonstrated the differential effects of key variables in different locations. However, there are significant differences in the biological characteristics and prognosis among these subtypes. This approach may still lead to the research conclusions being affected by tumor heterogeneity.

## 5 Conclusions

This study developed a new machine learning model based on multiple risk factors, which can effectively predict the prognosis of patients with biliary tract malignancies. This model may assist postoperative risk stratification and individualized adjuvant treatment decision-making.

## Data Availability

The original contributions presented in the study are included in the article/**Supplementary Material**. Further inquiries can be directed to the corresponding authors.

## Supplementary material

Supplementary Figure 1 Confusion matrices of different models in predicting 3-year prognosis. **(A)** Random Forest; **(B)** XGBoost; **(C)** KNN; **(D)** SVM; **(E)** LightGBM; **(F)** CatBoost.

Supplementary Figure 2 Confusion matrices of different models in predicting 5-year prognosis. **(A)** Random Forest; **(B)** XGBoost; **(C)** KNN; **(D)** SVM; **(E)** LightGBM; **(F)** CatBoost.

Supplementary Figure 3 SHAP analysis for quantifying and visualizing the contribution of each feature to the model in predicting 1-year prognosis. **(A)** The combination of SHAP bar chart and bee colony chart; **(B, C)** Force plots; **(D, E)** Waterfall plots.

Supplementary Figure 4 SHAP analysis for quantifying and visualizing the contribution of each feature to the model in predicting 3-year prognosis. **(A)** The combination of SHAP bar chart and bee colony chart; **(B, C)** Force plots; **(D, E)** Waterfall plots.
