# Supplementary material for: Construction and evaluation of machine learning models for postoperative prognosis prediction of biliary tract malignancies
Source: Front Oncol. 2026 Sep 15;16:1918078. doi: 10.3389/fonc.2026.1918078 (PMC13619383; doi:10.3389/fonc.2026.1918078)
Supplement: Supplementary file 1 [file Supplementaryfile1.docx]

**Supplementary File 1: Parameters of the six machine learning algorithms**

**1.RandomForest**

\# 创建随机森林分类器对象

rf = RandomForestClassifier(random_state=42)

\# 定义超参数的分布

param_distributions = {

    "n_estimators": np.arange(10, 201, 10),

    "max_depth": [None] + list(np.arange(3, 14)),

    "min_samples_split": np.arange(2, 11),

    "min_samples_leaf": np.arange(1, 6),

    "max_features": ["auto", "sqrt", "log2", None] + list(np.arange(1, X_train_12m.shape[1] + 1))

}

\# 创建随机搜索对象

random_search = RandomizedSearchCV(rf, param_distributions, n_iter=50, cv=5, random_state=42)

random_search.fit(X_train_12m,y_train_12m)

#获取最佳参数

best_params=random_search.best_params_

print("随机搜索的最佳参数：",best_params)

best_model = random_search.best_estimator_

**2.Xgboost**

\# 创建XGBoost分类器

xgb_clf = xgb.XGBClassifier(objective='binary:logistic', random_state=42)

\# 定义超参数网格

param_distributions = {

    'learning_rate': [0.01, 0.05, 0.1],               # 学习率

    'max_depth': [3, 6, 9],                            # 树的最大深度

    'n_estimators': [50, 100, 200],                    # 树的个数

    'subsample': [0.8, 0.9, 1.0],                      # 子样本比例

    'colsample_bytree': [0.8, 0.9, 1.0]                # 每棵树的列采样比例

}

\# 创建随机搜索对象

random_search = RandomizedSearchCV(xgb_clf, param_distributions, n_iter=50, cv=5, random_state=42)

random_search.fit(X_train_12m,y_train_12m)

#获取最佳参数

best_params=random_search.best_params_

print("随机搜索的最佳参数：",best_params)

best_model = random_search.best_estimator_

**3.KNN**

\# 创建KNN分类器对象

knn = KNeighborsClassifier()

\# 定义要搜索的超参数网格

param_distributions = {

    "n_neighbors": np.arange(1, 11),

    "weights": ["uniform", "distance"],

    "p": [1, 2]

}

\# 创建随机搜索对象

random_search = RandomizedSearchCV(knn, param_distributions, n_iter=50, cv=5, random_state=42)

random_search.fit(X_train_12m,y_train_12m)

#获取最佳参数

best_params=random_search.best_params_

print("随机搜索的最佳参数：",best_params)

best_model = random_search.best_estimator_

**4.Svm**

\# 创建SVM分类器对象

svc = SVC(random_state=42,probability=True)

param_distributions = {

    'C': [0.001,0.01,0.1,1,10,100],  # 正则化参数

    'gamma': [0.001,0.01,0.1,1,10,100],  # 核函数参数

    'kernel': ['linear', 'rbf', 'sigmoid'],  # 核函数类型    #注poly核函数跑的话特别消耗时间

}

\# 创建随机搜索对象

random_search = RandomizedSearchCV(svc, param_distributions, n_iter=10, cv=5, random_state=42,verbose=3)

random_search.fit(X_train_12m,y_train_12m)

#获取最佳参数

best_params=random_search.best_params_

print("随机搜索的最佳参数：",best_params)

best_model = random_search.best_estimator_

**5.Catboost**

\# 创建CatBoost分类器

catboost_clf = CatBoostClassifier(loss_function='MultiClass', random_state=42, cat_features=[],bootstrap_type='MVS')

\# 定义超参数网格

param_distributions = {

    'learning_rate': [0.01, 0.05, 0.1],              # 学习率

    'depth': [4, 6, 8],                               # 树的深度

    'iterations': [100, 200, 300],                    # 迭代次数（树的数量）

    'subsample': [0.8, 0.9, 1.0],                     # 子样本比例

    'colsample_bylevel': [0.8, 0.9, 1.0],             # 每一层的列采样比例

    'l2_leaf_reg': [3, 5, 7],                         # L2正则化的权重

}

\# 创建随机搜索对象

random_search = RandomizedSearchCV(catboost_clf, param_distributions, n_iter=50, cv=5, random_state=42)

random_search.fit(X_train_12m,y_train_12m)

#获取最佳参数

best_params=random_search.best_params_

print("随机搜索的最佳参数：",best_params)

best_model = random_search.best_estimator_

**6.Light gbm**

\# 创建LightGBM多分类分类器

lgbm_clf = lgb.LGBMClassifier(objective='binary', random_state=42)

\# 定义超参数网格

param_distributions = {

    'learning_rate': [0.01, 0.05, 0.1],          # 学习率

    'num_leaves': [31, 40, 50],                  # 每棵树的最大叶子数

    'max_depth': [-1, 5, 10],                     # 树的最大深度

    'n_estimators': [100, 200, 300],             # 迭代次数（树的数量）

    'subsample': [0.8, 0.9, 1.0],                 # 子样本比例

    'colsample_bytree': [0.8, 0.9, 1.0],          # 每棵树的列采样比例

    'reg_alpha': [0, 0.1, 0.5],                   # L1正则化

    'reg_lambda': [0, 0.1, 0.5],                  # L2正则化

}

\# 创建随机搜索对象

random_search = RandomizedSearchCV(lgbm_clf, param_distributions, n_iter=50, cv=5, random_state=42)

random_search.fit(X_train_12m,y_train_12m)

#获取最佳参数

best_params=random_search.best_params_

print("随机搜索的最佳参数：",best_params)

best_model = random_search.best_estimator_
