# Supplementary material for: Construction and evaluation of machine learning models for postoperative prognosis prediction of biliary tract malignancies
Source: Front Oncol. 2026 Sep 15;16:1918078. doi: 10.3389/fonc.2026.1918078 (PMC13619383; doi:10.3389/fonc.2026.1918078)
Supplement: Supplementary file 2 [file Supplementaryfile2.docx]

**Supplementary File 2-1: Cox proportional hazards regression analysis for intrahepatic cholangiocarcinoma**

| **Characteristics** | **B** | **SE** | **HR** | **CI** | **Z** | ***P*** |
| --- | --- | --- | --- | --- | --- | --- |
| Sex | 0.029 | 0.945 | 1.029 | 0.161-6.559 | 0.03 | 0.976 |
| Marital_status | -1.035 | 0.939 | 0.355 | 0.056-2.241 | -1.101 | 0.271 |
| Grade_Recode | 1.3 | 0.731 | 3.67 | 0.875-15.388 | 1.778 | 0.075 |
| Age | -0.427 | 0.939 | 0.653 | 0.104-4.107 | -0.455 | 0.649 |
| AJCC_Stage | -0.325 | 0.528 | 0.723 | 0.257-2.033 | -0.615 | 0.538 |
| Regional_nodes_examined | 0.009 | 0.039 | 1.009 | 0.934-1.09 | 0.225 | 0.822 |
| Regional_nodes_positive | -0.012 | 0.237 | 0.988 | 0.621-1.571 | -0.052 | 0.959 |
| LN_Sur_Scope | 0.45 | 0.997 | 1.569 | 0.222-11.068 | 0.452 | 0.652 |

**Supplementary File 2-2: Cox proportional hazards regression analysis for extrahepatic cholangiocarcinoma**

| **Characteristics** | **B** | **SE** | **HR** | **CI** | **Z** | ***P*** |
| --- | --- | --- | --- | --- | --- | --- |
| Marital_status | -0.28 | 0.071 | 0.755 | 0.657-0.868 | -3.949 | 0 |
| Grade_Recode | 0.149 | 0.05 | 1.16 | 1.053-1.279 | 2.996 | 0.003 |
| Sex | 0.124 | 0.069 | 1.132 | 0.989-1.297 | 1.799 | 0.072 |
| AJCC_Stage | 0.209 | 0.042 | 1.233 | 1.137-1.337 | 5.043 | 0 |
| Regional_nodes_examined | -0.015 | 0.004 | 0.985 | 0.977-0.994 | -3.412 | 0.001 |
| Age | 0.331 | 0.068 | 1.392 | 1.218-1.59 | 4.868 | 0 |
| Regional_nodes_positive | 0.088 | 0.011 | 1.092 | 1.067-1.116 | 7.638 | 0 |
| LN_Sur_Scope | -0.135 | 0.089 | 0.874 | 0.734-1.04 | -1.519 | 0.129 |

**Supplementary File 2-3: Cox proportional hazards regression analysis for gallbladder cancer**

| **Characteristics** | **B** | **SE** | **HR** | **CI** | **Z** | ***P*** |
| --- | --- | --- | --- | --- | --- | --- |
| Sex | 0.181 | 0.07 | 1.199 | 1.046-1.374 | 2.603 | 0.009 |
| Marital_status | -0.253 | 0.066 | 0.776 | 0.683-0.883 | -3.851 | 0 |
| Grade_Recode | 0.162 | 0.045 | 1.176 | 1.076-1.285 | 3.586 | 0 |
| Age | 0.443 | 0.068 | 1.558 | 1.363-1.78 | 6.507 | 0 |
| Regional_nodes_positive | 0.09 | 0.018 | 1.095 | 1.057-1.134 | 5.003 | 0 |
| Regional_nodes_examined | -0.004 | 0.004 | 0.996 | 0.989-1.003 | -1.014 | 0.311 |
| AJCC_Stage | 0.5 | 0.032 | 1.648 | 1.548-1.756 | 15.511 | 0 |
| LN_Sur_Scope | -0.29 | 0.075 | 0.748 | 0.646-0.867 | -3.865 | 0 |

**Supplementary File 2-4: Cox proportional hazards regression analysis for ampullary carcinoma**

| **Characteristics** | **B** | **SE** | **HR** | **CI** | **Z** | ***P*** |
| --- | --- | --- | --- | --- | --- | --- |
| Marital_status | -0.161 | 0.058 | 0.851 | 0.76-0.954 | -2.771 | 0.006 |
| Grade_Recode | 0.215 | 0.042 | 1.239 | 1.141-1.347 | 5.062 | 0 |
| Sex | 0.047 | 0.056 | 1.048 | 0.939-1.17 | 0.844 | 0.398 |
| AJCC_Stage | 0.364 | 0.037 | 1.439 | 1.34-1.547 | 9.944 | 0 |
| Regional_nodes_examined | -0.01 | 0.003 | 0.99 | 0.984-0.997 | -2.873 | 0.004 |
| Age | 0.508 | 0.056 | 1.662 | 1.489-1.855 | 9.05 | 0 |
| Regional_nodes_positive | 0.059 | 0.008 | 1.061 | 1.045-1.077 | 7.741 | 0 |
| LN_Sur_Scope | -0.315 | 0.108 | 0.73 | 0.591-0.902 | -2.917 | 0.004 |
