# Supplementary material for: Construction and evaluation of machine learning models for postoperative prognosis prediction of biliary tract malignancies
Source: Front Oncol. 2026 Sep 15;16:1918078. doi: 10.3389/fonc.2026.1918078 (PMC13619383; doi:10.3389/fonc.2026.1918078)
Supplement: Supplementary file 3 [file Supplementaryfile3.docx]

**Table :Collinearity Analysis of Candidate and Selected Variables.**

| **Variables** | **VIF (All candidate variables)** | **VIF (Selected features)** | **Inclusion in final model** |
| --- | --- | --- | --- |
| Age(65) | 1.037485208700671 | 1.012147770550479 | Yes |
| Sex | 1.075206708601275 | 1.082852798969825 | Yes |
| Marital status | 1.066200945440388 | 1.062440988679848 | Yes |
| Tumor Site | 1.166096570325168 | 1.146008033804442 | Yes |
| Grade Recode | 1.060418811637097 | 1.063021452149528 | Yes |
| AJCC Stage | 1.183794068526405 | 1.151097995381273 | Yes |
| Regional nodes examined | 1.632277948623009 | 1.614389146798281 | Yes |
| Regional nodes positive | 1.326389098922145 | 1.32114429029591 | Yes |
| LN Sur Scope | 1.515134193984216 | 1.528371773165717 | Yes |
| Tumor_size | 1.0282636220747 |  |  |
| Chemotherapy | 1.139426232358978 |  |  |
